# Clinical efficacy and safety of Danhong injection for the treatment of chronic heart failure: A protocol for systematic review

**DOI:** 10.1097/MD.0000000000019526

**Published:** 2020-04-03

**Authors:** Lihua Han, Chuan Wang, Dongfeng Yao, Bin Wang, Zhen Zhang, Jiping Liu

**Affiliations:** a Department of Pharmacology, College of Pharmacy, Shaanxi University of Chinese Medicine, Xianyang; b Key Laboratory of Pharmacodynamics and Material Basis of Chinese Medicine of Shaanxi Administration of Traditional Chinese Medicine; c Subject Innovation Team of Shaanxi University of Chinese Medicine; d Shaanxi Collaborative Innovation Center of Chinese Medicinal Resources Industrialization, Shaanxi University of Chinese Medicine, China.

**Keywords:** chronic heart failure, Danhong injection, meta-analysis, protocol, systematic review

## Abstract

**Background:**

Danhong injection (DHI) has been widely in the treatment of chronic heart failure (CHF) in China; however, there is not enough clinical evidence DHI for treating CHF.

**Methods:**

Two researchers will search literatures of DHI for CHF in databases. Extracted data are analyzed with Review Manager 5.3 software. The selected studies should be conducted quality evaluation, forest plots and funnel plots will be run by RevMan5.3.

**Results:**

This systematic review validates the clinical efficacy and safety of DHI in the treatment of CHF through the analysis of New York Heart Association functional classification, left ventricular ejection fraction, left ventricular end-diastolic dimension, cardiac output, brain natriuretic peptide, adverse events.

**Conclusions:**

This systematic review will be provided a rational clinical evidence to evaluate the effectiveness and safety of DHI for the treatment of CHF.

**Registration Number:**

PROSPERO CRD42019144686.

## Abstract 2

Supplemental Digital Content is available in the text

## 1 Introduction

Chronic heart failure (CHF) is one of the most common chronic diseases in the world and it has exerted great economic pressure on the public medical system.^[1,2]^ Patients with CHF experience a substantial reduction in their quality of life and increased risk for premature death. The drugs for the treatment of CHF that traditionally used include diuretics, angiotensin-converting enzyme inhibitors, beta-blockers, digoxin, etc. ^[3]^ However, These still have some problems in terms of clinical efficacy and safety. Danhong injection (DHI) is a Chinese drug used for coronary heart disease, angina pectoris, myocardial infarction, pulmonary heart disease, and cerebral infarction.^[4]^ DHI is composed of Danshen and Honghua, and the major active ingredients are tanshinone, salvianolic acid, safflower yellow pigment, phenol glycosides, and catechol. It has been widely and successfully used to treat patients with cardiovascular diseases for long time in China.^[5,6]^ However, more evidence is needed to prove its efficacy and safety if the drug can be approved in clinical guidelines. Currently, there are more and more randomized controlled trials (RCT) have reported the efficacy of DHI in the treatment of CHF. Therefore, we systematically evaluated the clinical efficacy and safety of DHI combined with basic Western medicine in the treatment of CHF.

## 2 Methods

### 2.1 Inclusion criteria

#### 2.1.1 Type of study

This meta-analysis includes all RCTs for CHF treated with DHI, regardless of region and country.

#### 2.1.2 Participants

According to World Health Organization^[7]^ and “Guidelines for the Diagnosis and Treatment of Chronic Heart Failure in China,”^[8]^ patients only include CHF and other non-CHF diseases are excluded.

#### 2.1.3 Type of intervention

In all studies, the treatment group, DHI will be intravenously instilled daily, on the basis of conventional therapy with CHF patients, while the control group was routinely treated with western medicine.

#### 2.1.4 Type of regions and gender

There are no languages, regional or gender restrictions in the inclusion study. At the same time, we will search for studies until December 2019.

#### 2.1.5 Outcomes

##### 2.1.5.1 Primary outcomes

The main results include New York Heart Association function classification, clinical total effective rate.

##### 2.1.5.2 Secondary outcomes

The secondary results of the analysis further confirmed the main results of the analysis, including left ventricular end-diastolic dimension, left ventricular ejection fraction, stroke volume, brain natriuretic peptide, N-terminal pro-brain natriuretic peptide, hypersensitive C-reactive protein and adverse events.

### 2.2 Search methods of studies

#### 2.2.1 Electronic searches

Two researchers will search China National Knowledge Infrastructure, Wanfang database, Chinese VIP Information, Chinese Biomedical Literature Database, PubMed, MEDLINE, The Cochrane Library, Web of Science, and other databases to evaluate the RCTs of DHI in the treatment of CHF

#### 2.2.2 Search strategy in electronic database

The search for PubMed will be performed using multiple combinations of the following terms: ^[9]^

main keywords: Danhong injection, chronic heart failure, randomized controlled trials

#1 (“heart failure, chronic” [MeSH Terms]) OR (“coronary heart disease∗” [Title/Abstract]) OR (“heart failure∗” [Title/Abstract]) OR (“chronic heart failure∗” [Title/Abstract]) OR (“coronary artery disease∗” [Title/Abstract])

#2 (“Danhong injection” [Title/Abstract]) OR (“Danhong, injection∗” [MeSH Terms]) OR (“Danhong Injectables∗” [Title/Abstract]) OR (“DHI injection∗” [Title/Abstract]) OR (“Danshen and Honghua, Injection∗” [Title/Abstract]) OR (“*S. miltiorrhiza* and *C. tinctorius* injection∗” [Title/Abstract]) OR (“*S. miltiorrhiza* and *C. tinctorius,* injection∗” [Title/Abstract]) OR (“*Salvia miltiorrhiza* and *Carthamus tinctorius* injection∗” [Title/Abstract]) OR (“*Salvia miltiorrhiza* and *Carthamus tinctorius*, injection∗” [Title/Abstract])

#3 (“Randomized, controlled trial” [MeSH Terms]) OR (“Randomized controlled trial∗” [Title/Abstract]) (“clinical study∗” [Title/Abstract]) OR (“Clinical Trial∗” [Title/Abstract]) OR (“Controlled study∗” [Title/Abstract]) OR (“Controlled Trial∗” [Title/Abstract])

#1 AND #2 AND #3

#### 2.2.3 Other resources

We searched for additional studies of reference lists of relevant primary studies, reviews, and conference journals.

### 2.3 Data collection and analysis

#### 2.3.1 Literature screening

All retrieved papers will be imported into an EndNote X9. Then duplicated papers will be excluded from the group.

**Figure 1:**
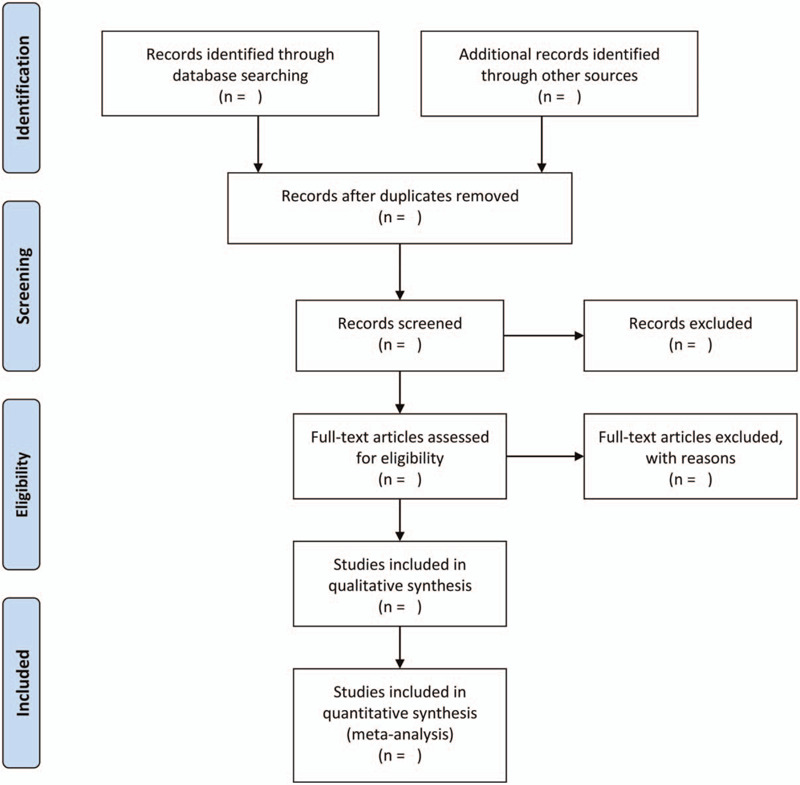
PRISMA flowchart of selection studies.

When screening literatures, 2 reviewers independently evaluated the title and abstract of the paper to exclude nonrelevant studies. Full-text studies will further screen studies that may meet the inclusion criteria, and in case of any disagreement, we will consult a third author that discuss into disagreement of selection studies. The details of the literature selection will be displayed in the PRISMA flowchart (Fig. 1).

#### 2.3.2 Data extraction

Two researchers independently screened the literature, the following data will be extracted from all the included studies: Study characteristics (author, year of publication, locations); Participants’ characteristics (age, gender, disease type, course of treatment, stage, interventions details, healing period, outcomes, and adverse events)

### 2.4 Assessment of methodological quality

The methodological quality of primary studies will be assessed by a revised tool devised for STROBE quality assessment. This has defined questions will be answered as a, b, c, d, e, and the score of each article will be calculated.

Selected literature can be divided into 7 considerations to evaluate the risk of bias, following the recommendations: random sequence generation method, allocation concealment, blinding of participants and personnel, blinding of outcome assessment, incomplete outcome data, selective reporting, and other offset sources. Each consideration is divided into 3 levels: “low risk,” “high risk,” and “unclear.” If two researchers do not reach an agreement, we will consult a third author that discuss into disagreement of selection studies. In addition, disagreements will be resolved by consensus.

### 2.5 Heterogeneity analysis

To investigate heterogeneity, we will include the study design (prospective or retrospective and year of publication) and population characteristics (gender, ethnicity, age, types of diseases, and stage distribution). The risk ratio was results of dichotomous variables with 95% confidence intervals (95%). The mean difference was the results of the continuous variables when outcomes were reported on the same scale. A heterogeneity test was used. If *P* > 0.1, the fixed effect model was used for meta-analysis. Otherwise, the random effect model was used. When *P* < 0.05, the difference between groups was statistically significant.

### 2.6 Publication bias

If there are more than 10 clinical studies, we should use a funnel plot to analyze whether it is symmetrical. Or some other methods, such as Begg rank correlation test and Egger linear regression test to evaluate publication bias. If necessary, we will also use STATA 12.0 software to evaluate the stability of the accompanying RCT.

### 2.7 Subgroup analysis

If subgroup analysis is needed, it will be conducted according to the age, gender, stage, grade, different treatment courses, different daily doses, people of different skin colors, and inclusion of differences in RCTs quality.

### 2.8 Sensitivity analysis

Sensitivity analysis is an important method used in meta-analysis to assess the robustness and reliability of results. The commonly used method is to eliminate each of the included studies one by one and then combine the effect quantities, change the inclusion of exclusion criteria or eliminate certain types of literature and then combine effect sizes.

## 3 Discussion

CHF is the end stage of various heart diseases and the 1-year fatality rate of patients with serious illness is as high as 50%.^[10]^ At present, the clinical treatment of CHF can improve the clinical symptoms of patients and enhance their quality of life,^[11]^ however, there has remained, nonetheless, a high residual burden of morbidity, and mortality in these patients.^[12]^ Traditional Chinese medicine has a long history and definite curative effect for treatment of chronic heart failure.^[13]^ At present, DHI and western medicine are widely used for the treatment of CHF in China.^[14,15]^ Therefore, we will conduct a meta-analysis that hopefully to provide proof of efficacy and safety of DHI for treating CHF. Due to the different methodological quality and dose of meta-analysis, the results obtained are not comprehensive. Therefore, we should have some reservations about the results of the system evaluation.

Appendix:

## Author contributions

**Conceptualization:** Chuan Wang, Zhen Zhang.

**Data curation:** Lihua Han, Jiping Liu, Bin Wang.

**Formal analysis:** Lihua Han, Dongfeng Yao.

**Funding acquisition:** Chuan Wang.

**Methodology:** Lihua Han, Jiping Liu.

**Software:** Lihua Han.

**Writing – original draft:** Lihua Han.

**Writing – review & editing:** Lihua Han, Chuan Wang.

Chuan Wang orcid: 0000-0002-8016-0113.

## Supplementary Material

Supplemental Digital Content
