# Supplementary material for: Clinical efficacy and safety of Danhong injection for the treatment of chronic heart failure: A protocol for systematic review
Source: Medicine (Baltimore). 2020 Apr 3;99(14):e19526. doi: 10.1097/MD.0000000000019526 (PMC7220450; doi:10.1097/MD.0000000000019526)
Supplement: Supplemental Digital Content [file medi-99-e19526-s001.docx]

**Supplementary Material**

**Search strategy used in PubMed database**

Danhong injection, chronic heart failure, randomized controlled trials

#1 (“heart failure, chronic” [MeSH Terms]) OR (“coronary heart disease∗” [Title/Abstract]) OR (“heart failure∗” [Title/Abstract]) OR (“chronic heart failure∗” [Title/Abstract]) OR (“coronary artery disease∗” [Title/Abstract])

#2 (“Danhong injection” [Title/Abstract]) OR (“Danhong, injection∗” [MeSH Terms]) OR (“Danhong Injectables∗” [Title/Abstract]) OR (“DHI injection∗” [Title/Abstract]) OR (“Danshen and Honghua, Injection∗” [Title/Abstract]) OR (“*S. miltiorrhiza* and *C. tinctorius* injection∗” [Title/Abstract]) OR (“*S. miltiorrhiza* and *C. tinctorius,* injection∗” [Title/Abstract]) OR (“*Salvia* *miltiorrhiza* and *Carthamus tinctorius* injection∗” [Title/Abstract]) OR (“*Salvia* *miltiorrhiza* and *Carthamus tinctorius*, injection∗” [Title/Abstract])

#3 (“Randomized, controlled trial” [MeSH Terms]) OR (“Randomized controlled trial∗” [Title/Abstract]) (“clinical study∗” [Title/Abstract]) OR (“Clinical Trial∗” [Title/Abstract]) OR (“Controlled study∗” [Title/Abstract]) OR (“Controlled Trial∗” [Title/Abstract])

#1 AND #2 AND #3
